# The influence of long-term treadmill exercise on bone mass and articular cartilage in ovariectomized rats

**DOI:** 10.1186/1471-2474-11-185

**Published:** 2010-08-20

**Authors:** Ting-Kuo Chang, Chang-Hung Huang, Chun-Hsiung Huang, Hsuan-Chiang Chen, Cheng-Kung Cheng

**Affiliations:** 1 Institute of Biomedical Engineering, National Yang Ming University, Taipei, Taiwan; 2 Department of Medical Research, Biomechanics Laboratory, Mackay Memorial Hospital, Taipei, Taiwan; 3 Department of Orthopaedic Surgery, Mackay Memorial Hospital, Taipei, Taiwan

## Abstract

**Background:**

Loss of bone quality and deterioration of articular cartilage are commonly seen after menopause. While exercise may protect against tissue degeneration, a clear link has yet to be established. The aim of the present study is to investigate the influence of long-term treadmill exercise on changes in bone mass and articular cartilage in ovariectomized rats.

**Methods:**

Sixty female Sprague-Dawley rats were randomly assigned to 4 groups: ovariectomized (OVX), ovariectomized plus treadmill exercise (OVX-RUN), treadmill exercise alone (RUN), and control (CON) groups. After 36 weeks, the following variables were compared among the 4 groups. Bone mass was evaluated by trabecular bone volume and bone mineral density (BMD). Articular cartilage in the knee joints was evaluated by histology analysis and a modified Mankin score.

**Results:**

Rats in the ovariectomized groups (OVX and OVX-RUN) had significantly lower BMD and bone mass than the non-ovariectomized rats (CON and RUN), indicating that exercise did little to preserve bone mass. However, the sedentary OVX group had a significantly worse modified Mankin score (7.7 ± 1.4) than the OVX-RUN group (4.8 ± 1.0), whose scores did not differ significantly from the other 2 non-operated groups. The articular cartilage in the sedentary OVX rats was relatively thinner, hypocellular, and had more clefts than in the other 3 groups.

**Conclusion:**

This study suggests that long-term exercise protects articular cartilage in OVX rats but does not retard the loss of bone mass seen in after menopause.

## Background

Osteoporosis is a systemic skeletal disease of unbalanced bone formation and resorption resulting in net bone loss and reduced bone strength. It is common in post-menopausal women [1,2]. Previous studies have shown that exercise improve bone mass, density and strength, and thus may help to prevent osteoporosis [3-7]. Exercise programs, such as running on a treadmill, have been widely used to study the effects of physical activity on osteoporosis and bone mass. Though most studies have focused on the effect of exercise on bone formation or changes in bone mineral density (BMD), changes in cartilage after menopause have been rarely assessed. One study in sedentary aging rats indicated that ovariectomy may accelerate cartilage degeneration [8]. Another study in rabbits showed that osteoporosis increased the severity of cartilage damage in the anterior cruciate ligament resection model [9]. However, the latter study involved surgically induced trauma and thus did not closely resemble natural degeneration of human cartilage. It is important to evaluate cartilage changes using a well-established animal model that more closely resembles physiologic changes seen in humans. In addition, if exercise indeed has a prophylactic role, it should be included the study design.

Ovariectomized (OVX) rats are the most commonly used animal model for studying osteoporosis in menopausal women. The bone loss induced by ovariectomy in rats shares many clinical characteristics with human post-menopausal bone loss [10,11]. To the best of our knowledge, even when exercise was used in such animal studies, the duration was for only 6 to 16 weeks [1,4,5,12-14]. A longer study duration would be preferable to investigate the effect of treadmill exercise on bone mass and articular cartilage in OVX rats.

We hypothesized that OVX rats exercising long-term would have less cartilage degeneration than rats that did not exercise. The aim of this longitudinal animal study therefore was to determine the effects of long-term treadmill exercise on bone mass and degenerative change of knee joint cartilage in OVX rats.

## Methods

### Animals and exercise program

**Figure 1:**
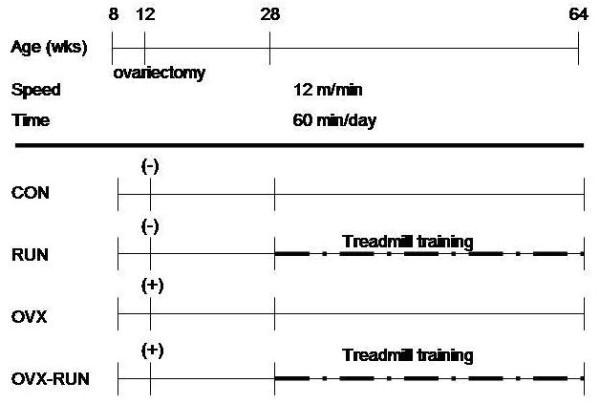
**Experimental protocol, ovariectomy was performed in rats at the age of 12 weeks, and treadmill exercise started at 28 weeks**. (+) indicates ovariectomy and (-) indicates no ovariectomy.

Sixty 8-week-old female Sprague-Dawley rats (weight 229 ± 9 g) were purchased from BioLASCO Taiwan Co., Ltd. (Taipei, Taiwan). The animals were housed separately in single cages (30 × 28 × 20 cm^3^) and kept in a controlled environment (23 ± 1°C, 12 h light-dark cycle) with free access to food and water. After four weeks of acclimation to the new environment, the rats were randomly assigned to four groups (15 rats per group): OVX, OVX plus exercise (OVX-RUN), exercise (RUN), and control (CON). At 12 weeks of age, the OVX groups (OVX and OVX-RUN) underwent bilateral ovariectomy using the dorsal approach under anesthesia (intramuscular ketamine, 8.7 mg/100 g of body weight and xylazine, 1.3 mg/100 g of body weight). The CON and RUN groups did not undergo any surgical procedure. Sixteen weeks after surgery, the RUN and OVX-RUN rats commenced 36 weeks of exercise on a motor-driven treadmill (Model T510, DRI Co., Taoyuan, Taiwan). The speed was set at 12 m/min and the rats ran on the treadmill for 60 minutes each day on 5 days of every week. This intensity is considered a moderate level of exercise [4]. The animals in the OVX and CON groups were kept sedentary throughout the 36-week study period (Figure 1). The study protocol was approved by the Institutional Animal Care and Use Committee of our institution.

### Bone mineral density measurement

Rats were anesthetized in the same manner as described above for ovariectomy and placed in a prone position. BMD was determined in the lumbar spine with the use of dual energy X-ray absorptiometry (Norland XR-36, USA). The scan was set to an ultra high-resolution scan mode using the manufacturer's software. The lumbar BMD was expressed as the mean value obtained from L3, L4, and L5 vertebral bodies.

### Tissue preparation

After the BMD measurements were completed, all rats were killed with CO_2 _anesthesia. The uterus was retrieved from the abdominal cavity of each rat and weighed. A lower limb was also removed. Tissue samples were taken from the knee joint and proximal tibia and fixed in natural buffer formalin for 48 h and decalcified in 10% EDTA (PH 7.2) at 4°C for 5 weeks. The samples were then dehydrated in graded ethanol, cleaned in xylene, and embedded in paraffin. Serial longitudinal sections (6 μm) were cut with a rotary microtome (KD 2508, KEDEE, Jinhua, China) and mounted on saline-coated glass slides (DAKO North America, Inc., CA, USA). Before staining, the sections were deparaffinized in 100% xylene and rehydrated in graded ethanol.

### Histology

Tissue sections of the knee joints were stained with Masson trichrome and for tartrate-resistant acid phosphatase (TRAP) using commercially available kits (Sigma Chemical Co., St Louis, USA). The articular cartilage was evaluated using a modified Mankin score [15,16], examining the surface, cellularity, staining, and cloning. Each characteristic is scored from 0 to 3, with higher scores reflecting worse degenerative change. The highest possible score was 12 (Table 1). This scoring method consists of four different parameters with a maximum of 3 points each. Thus, 12 points represents the worse degenerative change of cartilage and zero signifies no change. Histomorphometric measurements of the cancellous bone of the proximal tibia were performed semi-automatically with an Olympus BX 40 microscope and an Olympus DP-70 digital camera (Olympus, Tokyo, Japan). Data were further analyzed using commercially available software (Multi Gauge v2.1, Fuji film Co., Tokyo, Japan). The trabecular bone volume in the metaphysis of the proximal tibia was calculated according to Parfitt's method as BV/TV, or bone volume (BV) divided by tissue volume (TV) [4,17]. The histologic evaluation was performed independently by two authors (Chang and Chen) who were blinded to the group each rat was in when the examination was done.

**Table 1:** Modified Mankin score for evaluating the severity of cartilage damage.

Variable	Score
Surface	0 = normal
	1 = irregular
	2 = fibrillated/vaculoated
	3 = clefts/erosion
Cellularity	0 = normal
	1 = slight decrease in chondrocytes
	2 = marked decrease in chondrocytes
	3 = no cells
Staining	0 = normal
	1 = little decrease in staining
	2 = marked decrease in staining
	3 = no staining
Cloning	0 = normal
	1 = occasional doublets
	2 = doublets and triplets
	3 = multiple nested cells

Note: The maximal score is 12 points. Higher score means higher degree of cartilage degeneration.

### Statistical analysis

Data are expressed as the mean values and standard deviations of the different continuous variables. The Mann-Whitney U-test was used to compare the results among the 4 groups. A *P *value of less than 0.05 was considered to be significant.

## Results

### Weight of uterus

The mean uterine weights in the CON and RUN groups were 0.912 ± 0.195 g and 0.876 ± 0.208 g, significantly higher (*P *< 0.001) than in the OVX and OVX-RUN groups (0.119 ± 0.021 g and 0.118 ± 0.018 g). There were no statistically significant differences between the CON and RUN groups. Marked atrophy of the uterus in the ovariectomy groups was noted in this study.

### Bone mineral density

**Figure 2:**
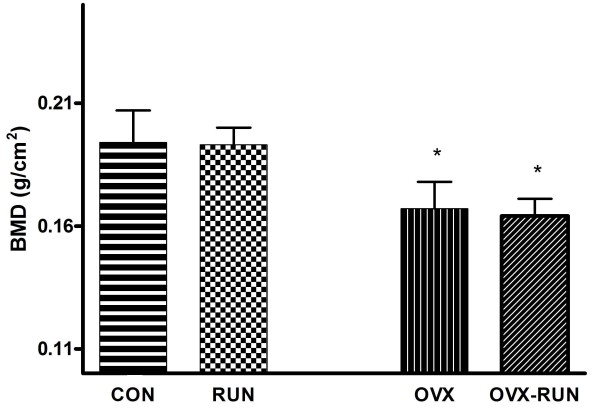
**Bone mineral density (BMD) after 36 weeks of exercise**. Data are mean ± SD. *P < 0.05 compared to non-ovariectomized groups (CON and RUN).

The mean BMD following euthanasia was 0.194 ± 0.013 g/cm^2 ^(CON), 0.193 ± 0.007 g/cm^2 ^(RUN), 0.167 ± 0.011 g/cm^2 ^(OVX) and 0.168 ± 0.007 g/cm^2 ^(OVX-RUN), respectively (Figure 2). Rats in the groups without an ovariectomy (CON and RUN) had a significantly higher BMD than in the groups with an ovariectomy (*P *< 0.05). On the other hand, rats in the groups without treadmill exercise (CON, OVX) have no significant difference in BMD when compared to the groups with exercise.

### Histology

**Figure 3:**
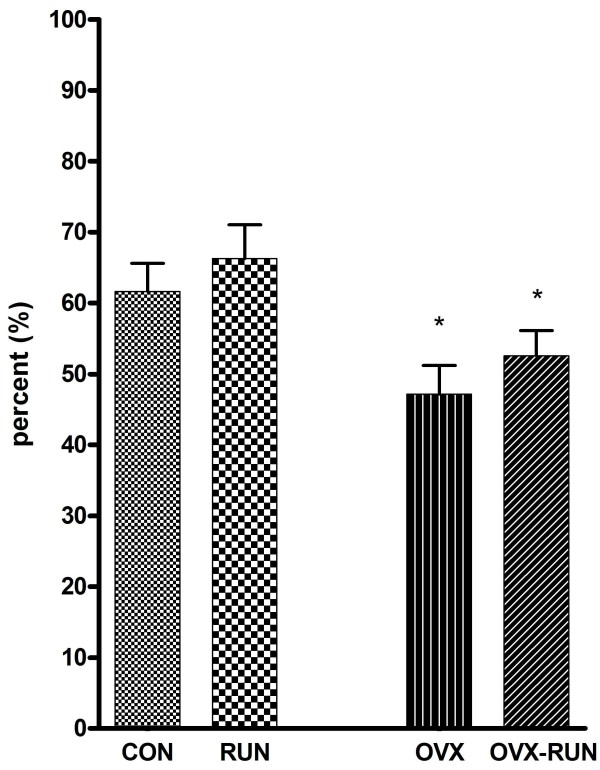
**The results of trabecular bone volume (BV/TV) in the proximal tibia**. *P < 0.05 compared to non-ovariectomized groups (CON and RUN).

**Figure 4:**
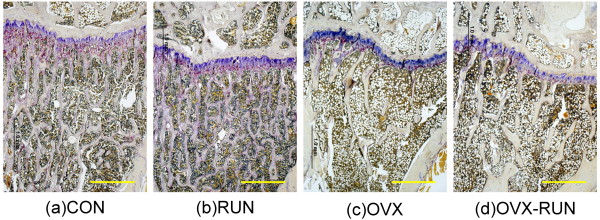
**Photomicrographs of the proximal tibia of rats (TRAP stain, ×4)**. The ovariectomized groups (OVX-c, OVX-RUN-d) appeared less trabecular numbers and significantly increased porosity of marrow cavity than CON(a) and RUN(b) groups. Scale bars is = 1 mm.

The results of trabecular bone volume (BV/TV) in proximal tibia are shown in Figure 3. The mean BV/TV in the RUN group was 66.3% and in the CON group 61.6%, both of which were significantly higher than in the OVX-RUN (52.6%) or OVX (47.1%) group (*P *< 0.05). There was no significant statistically difference between the RUN and CON rats. The porosity of the proximal tibial metaphysis was highest in the OVX group. It was slightly lower in the OVX-RUN group, but the difference was not statistically significant. Both RUN and CON groups had higher trabecular numbers and trabecular connections as well as less porosity of the marrow cavity than the 2 OVX groups (Figure 4).

**Figure 5:**
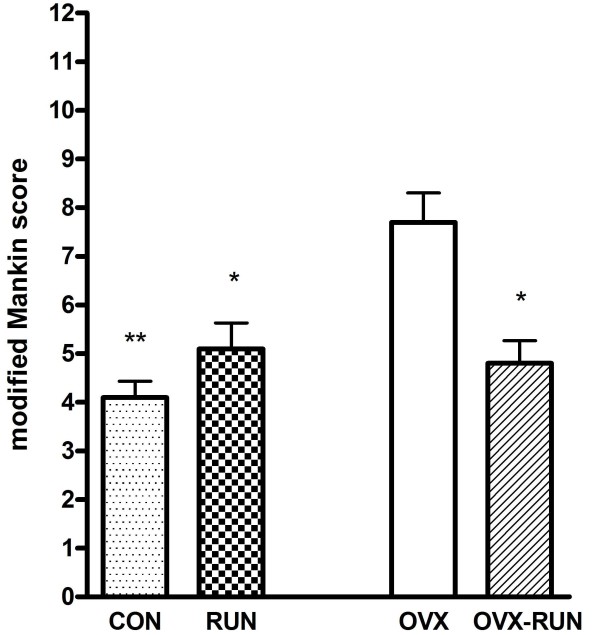
**The results of modified Mankin scores**. Data are mean ± SD. The OVX group has statistically higher mean scores when compared to other groups (*P < 0.05, ** *P *< 0.01).

**Figure 6:**
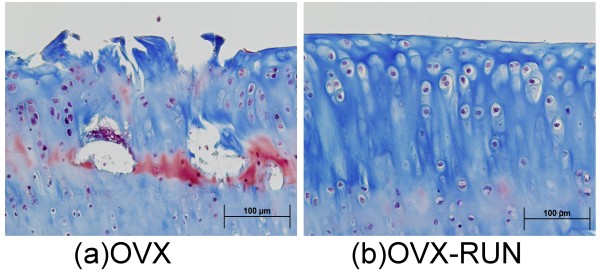
**Articular cartilage (Masson trichrome stain, × 20)**. Surface fibrillation, thinner articular cartilage, decreased staining and cleft formation are present in (a) a sedentary ovariectomized rat (OVX) but not in (b) an exercised ovariectomized rat (OVX-RUN).

The mean Mankin score in the OVX group was 7.7 ± 1.4, significantly higher than in the other 3 groups (OVX-RUN, 4.8 ± 1.0; RUN, 5.1 ± 1.2; CON, 4.1 ± 0.7; *P *< 0.05). The Mankin scores did not differ among those 3 groups (Figure 5). Unexercised rats in the OVX group had relatively thinner joint cartilage, lower cellularity, and cleft formation (Figure 6a) compared with the articular cartilage of OVX-RUN rats (Figure 6b), indicating that exercise was associated with better preservation of articular cartilage.

## Discussion

The ovariectomized rat model has been recognized as a stable and common animal model to study postmenopausal bone loss [10,11]. The effects of treadmill exercise on bone mass have been studied extensively, but there is a lack of evidence about its influence on articular cartilage change. In this study, we used an ovariectomized rat model to evaluate the influence of long-term exercise on changes in cartilage and bone mass.

Results of the BMD after 36 weeks of exercise in the OVX and OVX-RUN groups were significantly lower than the CON rats (16.2% in OVX and 15.5% in OVX-RUN) and the RUN group (15.6% in OVX and 14.9% in OVX-RUN). Similar results were reported by Flieger et al. [18]. They demonstrated bone loss of about 13.8% in the distal femur and 12.1% in the proximal tibia of ovariectomized rats compared to control ones. In addition, one recent study showed a 9.5% decrease of BMD in rats after receiving ovariectomy 32 weeks later compared to rats in control group [19]. Our results of decreased BMD in ovariectomized rats were consistent with these findings. Uterus weight following euthanasia in the CON and RUN rats were significantly heavier than that of the ovariectomy groups (7.7 and 7.4 times, respectively). The results were similar to a previous study showed an ovariectomy induced significant regression of the uterus compared with control group [8]. This may be caused by estrogen deficiency after menopause.

Iwamoto et al. demonstrated that there was no significant effect on lumbar bone mass after moderate exercise in ovariectomized rats [4]. They suggested that the response of the cancellous bone to exercise differs according to the mechanical loading of the bone. In addition, a longer treadmill study (13 weeks and 28 weeks) conducted by Gala et al. showed significant loss of lumbar BMD after OVX (19.5% and 30.3%) compared to the control group. However, exercise alone had no significantly influence on the lumbar BMD of OVX rats following 28 weeks of experimentation [20]. Similarly, our long-term (56 weeks experiment with a total of 36 weeks of treadmill exercise) moderate exercise study could not show significantly elevated BMD in OVX rats. This finding may be correlated with a previous study that the positive influence of treadmill exercise on BMD is site specific [14], with weight-bearing sites predominating [21].

The trabecular bone volume in the RUN group significantly increased by about 40.8% and 26.0% compared to that of OVX and OVX-RUN group, while the CON group saw increases of 30.8% and 17.1% respectively. The cancellous BV/TV of the tibia in the OVX-RUN group is slightly increased about 11.6% than the OVX group, but this didn't make a significantly statistical difference. These results further confirmed our BMD data that ovariectomy can induce poor architecture of bone trabeculae and exercise alone has no significant beneficial effect on bone mass.

The early stage of cartilage degeneration in humans is difficult to assess at present; therefore, using an animal model to study the cartilage change after menopause is more efficacious. In this study, we used a modified Mankin score method to evaluate the cartilage degeneration in different groups. The score was significantly higher in OVX rats than other groups (+3.6 points vs. CON, +2.9 points vs. OVX-RUN and +2.6 points vs. RUN, respectively). This is consistent with Otterness et al.'s study [22]. They determined the effects of articular cartilage and synovial fluid on the development of cartilage degeneration in hamsters with or without 3 months of wheel running exercise. Extensive fibrillation of the articular surface was seen in the sedentary group. This may have been caused by lower proteoglycan content and lower synovial fluid volume [22]. Our present results observed that a relatively thinner cartilage layer, decreased matrix staining, hypocellularity and cleft formation in the photomicrograph of the OVX group. This supports that sedentary life in post-menopausal rats may induce cartilage degeneration change. Long-term moderate treadmill exercise seems to have a chondroprotective effect on OVX rats in our study. The reasons why running exercise can influence the cartilage change after ovariectomy need further clarified.

Cartilage degeneration after ovariectomy has been studied extensively before. In a systematic review, Sniekers et al. analyzed the results of animal studies of osteoarthritic change after ovariectomy and of the effects of estrogen treatment [23]. They found that 11 out of 14 studies showed a detrimental effect of ovariectomy on articular cartilage in sexually mature animals. However, the results of estrogen treatment were conflicting across a number of studies. In part, they found substantial differences in study design, so that the findings were difficult to compare. However, they noted that OVX-related hormonal changes involve more than a simple estrogen deficit, since the levels of progesterone, follicle-stimulating hormone, or luteinizing hormone change as well. Perhaps OVX-induced articular damage is a more complex process than we realize, which might explain why estrogen treatment is not uniformly successful in protecting against OVX-induced articular degeneration. The true mechanism of menopausal cartilage degeneration may be multi-factorial and needs further investigation.

There are several limitations to this study. First, we reported lumbar BMD instead of the distal femur or the proximal tibia because higher variation of BMD was found on the femur than that of spine. The possible reason resulting in higher variation for femoral BMD might be smaller dimension of femoral bone. Central skeleton (Lumbar BMD) would help eliminate the variation. In addition, the data of trabecular bone mass further proved that relative osteoporosis was seen after ovariectomy, which also correlates with the BMD result of spine. Secondly, it is noted that estrogen may have a protective effect on OA [9], and estrogen deficiency could result in cartilage degeneration [8]. However, we didn't include estrogen analysis in this study but instead used uterus weight to measure the effect of estrogen indirectly. Thirdly, the current study found that moderate exercise seems to play a positive role on chondroprotective effect in ovariectomized rats but seems no obviously changes on the bone tissue. Except for slightly increase of the ratio of BV/TV (11.6%) was found in the OVX-RUN group when compared to the OVX group. Further detailed assessments will be helpful to justify the realistic mechanism of exercise on bone mass. Finally, the scoring of articular damage is somewhat subjective and has an individual bias; therefore, this analysis was conducted by two independent observers to ensure repeatability and consistency.

## Conclusions

In summary, this long-term study suggests that treadmill running exercise may contribute a certain degree of protection against cartilage degeneration in rats after ovariectomy. Long-term exercise could not increase the trabecular bone volume and lumbar bone mineral density in our animal study.

## Competing interests

The authors declare that they have no competing interests.

## Authors' contributions

TC and CK designed and initiated the study and participated in the interpretation of results and manuscript development. CH participated in the interpretation of results and manuscript development. TC and HC performed the statistical analysis. TC and CH participated in the analysis and discussion of results and contributed to manuscript development. All authors read and approved the final manuscript.

## Pre-publication history

The pre-publication history for this paper can be accessed here:

http://www.biomedcentral.com/1471-2474/11/185/prepub
